# 2-Chloro-*N*-[2-(2-fluoro­benzo­yl)-4-nitro­phen­yl]-*N*-methyl­acetamide

**DOI:** 10.1107/S1600536811034969

**Published:** 2011-08-31

**Authors:** B. P. Siddaraju, Jerry P. Jasinski, James A. Golen, H. S. Yathirajan, C. R. Raju

**Affiliations:** a Department of Studies in Chemistry, University of Mysore, Manasagangotri, Mysore 570 006, India; b Department of Chemistry, Keene State College, 229 Main Street, Keene, NH 03435-2001, USA; c Department of Chemistry, PES College of Science, Mandya 571 401, India

## Abstract

The title compound, C_16_H_12_ClFN_2_O_4_, crystallizes with two mol­ecules in the asymmetric unit in which the dihedral angles between the mean planes of the two benzene rings are 65.1 (7) and 65.6 (6)°. In each mol­ecule, the nitro group displays rotational disorder over two orientations in a 0.503 (11):0.497 (11) ratio and the Cl atom is disordered in a 0.432 (5):0.568 (5) ratio. In one mol­ecule, the F atoms is statistically disordered over two positions. The crystal packing features weak inter­molecular C—H⋯O and C—H⋯Cl inter­actions, which form a layered network.

## Related literature

For anti-anaphylactic and disease-related agents, see: Evans *et al.* (1987 ▶). For an inter­mediate in the synthesis of flunitra­zepam (systematic name: 6-(2-fluoro­phen­yl)-2-methyl-9-nitro-2,5-diaza­bicyclo­[5.4.0]undeca-5,8,10,12-tetraen-3-one), see: Malanciuc *et al.* (2009 ▶). For related structures, see: Dutkiewicz *et al.* (2010 ▶); Jasinski *et al.* (2009 ▶); Khan *et al.* (2010 ▶); Malathy Sony *et al.* (2005*a* ▶,*b* ▶); Prasanna & Guru Row (2000 ▶). For standard bond lengths, see Allen *et al.* (1987 ▶).
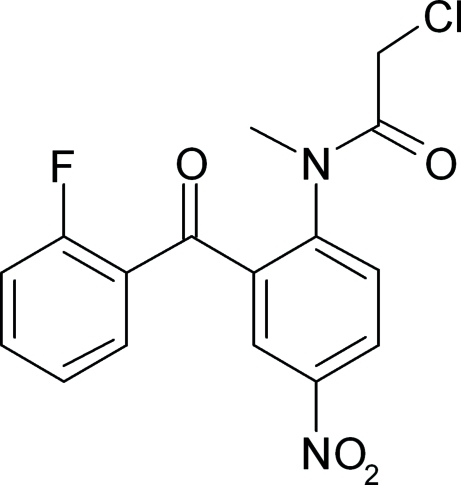


## Experimental

### 

#### Crystal data

C_16_H_12_ClFN_2_O_4_
*M* *_r_* = 350.73
Triclinic, 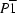

*a* = 8.1339 (6) Å
*b* = 10.9639 (8) Å
*c* = 17.8690 (11) Å
α = 81.251 (6)°
β = 82.239 (6)°
γ = 87.937 (6)°
*V* = 1560.38 (19) Å^3^
*Z* = 4
Mo *K*α radiation
μ = 0.28 mm^−1^
*T* = 200 K
0.24 × 0.16 × 0.12 mm

#### Data collection

Oxford Diffraction Xcalibur Eos Gemini diffractometer
Absorption correction: multi-scan (*CrysAlis RED*; Oxford Diffraction, 2010 ▶) *T*_min_ = 0.936, *T*_max_ = 0.967
13030 measured reflections
6373 independent reflections
3774 reflections with *I* > 2σ(*I*)
*R*_int_ = 0.041

#### Refinement

*R*[*F* ^2^ > 2σ(*F* ^2^)] = 0.066
*wR*(*F* ^2^) = 0.172
*S* = 1.03
6373 reflections
448 parameters
16 restraints
H-atom parameters constrained
Δρ_max_ = 0.32 e Å^−3^
Δρ_min_ = −0.36 e Å^−3^

### 

Data collection: *CrysAlis PRO* (Oxford Diffraction, 2010 ▶); cell refinement: *CrysAlis PRO*; data reduction: *CrysAlis RED* (Oxford Diffraction, 2010 ▶); program(s) used to solve structure: *SHELXS97* (Sheldrick, 2008 ▶); program(s) used to refine structure: *SHELXL97* (Sheldrick, 2008 ▶); molecular graphics: *SHELXTL* (Sheldrick, 2008 ▶); software used to prepare material for publication: *SHELXTL*.

## Supplementary Material

Crystal structure: contains datablock(s) global, I. DOI: 10.1107/S1600536811034969/im2312sup1.cif

Structure factors: contains datablock(s) I. DOI: 10.1107/S1600536811034969/im2312Isup2.hkl

Supplementary material file. DOI: 10.1107/S1600536811034969/im2312Isup3.cml

Additional supplementary materials:  crystallographic information; 3D view; checkCIF report

## Figures and Tables

**Table 1:** Hydrogen-bond geometry (Å, °)

*D*—H⋯*A*	*D*—H	H⋯*A*	*D*⋯*A*	*D*—H⋯*A*
C32—H32*A*⋯O1	0.98	2.48	3.383 (4)	154
C29—H29⋯O5^i^	0.95	2.50	3.194 (5)	130
C28—H28⋯Cl1*A*^ii^	0.95	2.74	3.543 (6)	143
C17—H17*A*⋯O1	0.99	2.38	3.348 (4)	165
C16—H16*B*⋯O2^iii^	0.98	2.47	3.45 (1)	176
C7—H7⋯O7^iv^	0.95	2.37	3.31 (2)	173

Symmetry codes: (i) 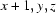; (ii) 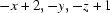; (iii) 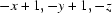; (iv) 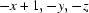.

**Fig. 1.**
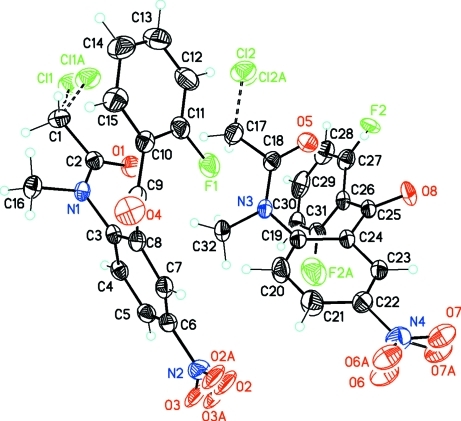
Molecular structure of the title molecule showing two molecules in the asymmetric unit, displacement ellipsoids are depicted on the 30% probability level. Dashed lines represent disordered atoms.

**Fig. 2.**
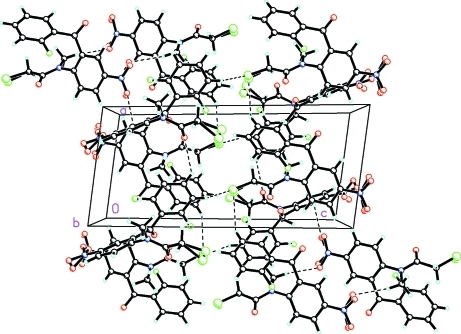
Packing diagram of the title compound viewed down the b axis. Dashed lines indicate weak intermolecular C—H···O and C—H···Cl interactions.

## supplementary crystallographic information

### Comment

Benzophenone and related compounds have been reported to act as antiallergic, anti-inflammatory, antiasthmatic, antimalarial, antimicrobial and antianaphylactic agents (Evans *et al.*, 1987). The title compound is an intermediate in the synthesis of certain anxiolytic, anticonvulsant and sedative drugs and is also used as an intermediate to synthesize flunitrazepam, which is used as a potent hypnotic and powerful sedative, anticonvulsant, anxiolytic, amnestic, and skeletal muscle relaxant drug (Malanciuc *et al.*, 2009). The crystal structures of 2-chloroacetamido-5-chloro-2'-fluorobenzophenone (Prasanna & Guru Row, 2000), *N*-(2-benzoyl-4-chlorophenyl)-2- chloroacetamide (Malathy Sony *et al.*, 2005*a*), 2-methoxy-5-methylphenyl phenyl ketone (Malathy Sony *et al.*, 2005*b*), 2-amino-5-nitrophenyl 2-chlorophenyl ketone (Jasinski *et al.*, 2009), *N*-[4-chloro-2-(2-chlorobenzoyl)phenyl]acetamide (Khan *et al.*, 2010) and 2-chloro-*N*-[4-chloro-2-(2-chlorobenzoyl)phenyl]acetamide (Dutkiewicz *et al.*, 2010) have been reported. In view of the importance of the title compound, C_16_H_12_N_2_O_4_ClF, the crystal structure of (I) is reported.

The title compound, C_16_H_12_N_2_O_4_ClF, crystallizes with two molecules in the asymmetric unit (Fig. 1). The dihedral angle between the mean planes of the two benzene rings is 65.1 (7)° and 65.6 (6)°, respectively. Bond lengths are in normal ranges (Allen *et al.*, 1987). The two nitro groups display rotational disorder over two positions in a ratio of 0.503 (11) : 0.497 (11). In addition, disorder is observed concerning both chlorine atoms in a ratio of 0.432 (5) : 0.568 (5). One of the fluorine atoms (F2) is statistically disordered (0.50 (0)) over two positions (F2 and F2A). The crystal packing is realized by weak intermolecular C—H···O and C—H···Cl interactions forming a supermolecular 2-D network (Fig. 2).

### Experimental

The title compound was obtained as a gift sample from R.*L*. Fine Chem., Bangalore, India. The compound was recrystallized from acetone (m.p.: 387–389 K).

### Refinement

The two nitro groups are rotationally disordered over two positions [O2 & O3 (0.503 (11), O2A & O3A (0.497 (11)) and O6 & O7 (0.503 (11), O6A & O7A (0.497 (11)]. In addition, disorder is observed concerning Cl1 & Cl2 (0.432 (5)) and Cl1A & Cl2A (0.568 (5)). Moreover, F2 and F2A are disordered (0.50 (0)) over two positions. All H atoms were placed in their calculated positions and then refined using the riding model with C–H lengths of 0.95 Å (CH), 0.99 Å (CH_2_) or 0.98 Å (CH_3_). The isotropic displacement parameters for these atoms were set to 1.19–1.20 (CH, CH_2_) or 1.50 (CH_3_) times *U*_eq_ of the parent atom.

### Crystal data

**Table:**

C_16_H_12_ClFN_2_O_4_	*Z* = 4
*M**_r_* = 350.73	*F*(000) = 720
Triclinic, *P*1	*D*_x_ = 1.493 Mg m^−^^3^
Hall symbol: -P 1	Mo *K*α radiation, λ = 0.71073 Å
*a* = 8.1339 (6) Å	Cell parameters from 2929 reflections
*b* = 10.9639 (8) Å	θ = 3.1–32.4°
*c* = 17.8690 (11) Å	µ = 0.28 mm^−^^1^
α = 81.251 (6)°	*T* = 200 K
β = 82.239 (6)°	Block, colorless
γ = 87.937 (6)°	0.24 × 0.16 × 0.12 mm
*V* = 1560.38 (19) Å^3^	

### Data collection

**Table:**

Oxford Diffraction Xcalibur Eos Gemini diffractometer	6373 independent reflections
Radiation source: Enhance (Mo) X-ray Source	3774 reflections with *I* > 2σ(*I*)
graphite	*R*_int_ = 0.041
Detector resolution: 16.1500 pixels mm^-1^	θ_max_ = 26.4°, θ_min_ = 3.1°
ω scans	*h* = −10→10
Absorption correction: multi-scan (*CrysAlis RED*; Oxford Diffraction, 2010)	*k* = −12→13
*T*_min_ = 0.936, *T*_max_ = 0.967	*l* = −22→21
13030 measured reflections	

### Refinement

**Table:**

Refinement on *F*^2^	Primary atom site location: structure-invariant direct methods
Least-squares matrix: full	Secondary atom site location: difference Fourier map
*R*[*F*^2^ > 2σ(*F*^2^)] = 0.066	Hydrogen site location: inferred from neighbouring sites
*wR*(*F*^2^) = 0.172	H-atom parameters constrained
*S* = 1.03	*w* = 1/[σ^2^(*F*_o_^2^) + (0.0665*P*)^2^ + 0.3335*P*] where *P* = (*F*_o_^2^ + 2*F*_c_^2^)/3
6373 reflections	(Δ/σ)_max_ = 0.012
448 parameters	Δρ_max_ = 0.32 e Å^−^^3^
16 restraints	Δρ_min_ = −0.36 e Å^−^^3^

### Special details

**Table:**

Geometry. All e.s.d.'s (except the e.s.d. in the dihedral angle between two l.s. planes) are estimated using the full covariance matrix. The cell e.s.d.'s are taken into account individually in the estimation of e.s.d.'s in distances, angles and torsion angles; correlations between e.s.d.'s in cell parameters are only used when they are defined by crystal symmetry. An approximate (isotropic) treatment of cell e.s.d.'s is used for estimating e.s.d.'s involving l.s. planes.
Refinement. Refinement of *F*^2^ against ALL reflections. The weighted *R*-factor *wR* and goodness of fit *S* are based on *F*^2^, conventional *R*-factors *R* are based on *F*, with *F* set to zero for negative *F*^2^. The threshold expression of *F*^2^ > σ(*F*^2^) is used only for calculating *R*-factors(gt) *etc*. and is not relevant to the choice of reflections for refinement. *R*-factors based on *F*^2^ are statistically about twice as large as those based on *F*, and *R*- factors based on ALL data will be even larger.

### Fractional atomic coordinates and isotropic or equivalent isotropic displacement parameters (Å^2^)

**Table:**

	*x*	*y*	*z*	*U*_iso_*/*U*_eq_	Occ. (<1)
C1	0.6305 (4)	0.5284 (3)	0.37754 (17)	0.0528 (9)	
H1A	0.5092	0.5222	0.3930	0.063*	
H1B	0.6554	0.6170	0.3618	0.063*	
C2	0.6740 (4)	0.4643 (3)	0.30868 (17)	0.0397 (7)	
C3	0.6349 (4)	0.4613 (2)	0.17778 (16)	0.0357 (7)	
C4	0.7948 (4)	0.4601 (3)	0.13956 (18)	0.0409 (7)	
H4	0.8838	0.4889	0.1612	0.049*	
C5	0.8253 (4)	0.4175 (3)	0.07052 (18)	0.0447 (8)	
H5	0.9352	0.4142	0.0449	0.054*	
C6	0.6941 (4)	0.3797 (3)	0.03924 (17)	0.0444 (8)	
C7	0.5340 (4)	0.3788 (3)	0.07590 (17)	0.0431 (8)	
H7	0.4455	0.3525	0.0529	0.052*	
C8	0.5044 (4)	0.4168 (3)	0.14665 (17)	0.0379 (7)	
C9	0.3307 (4)	0.4051 (3)	0.18774 (19)	0.0448 (8)	
C10	0.3030 (4)	0.3575 (3)	0.27043 (19)	0.0434 (8)	
C11	0.3959 (4)	0.2618 (3)	0.3054 (2)	0.0508 (9)	
C12	0.3721 (5)	0.2190 (4)	0.3814 (2)	0.0683 (11)	
H12	0.4374	0.1526	0.4030	0.082*	
C13	0.2500 (6)	0.2752 (4)	0.4261 (2)	0.0799 (13)	
H13	0.2326	0.2486	0.4797	0.096*	
C14	0.1531 (5)	0.3689 (4)	0.3946 (3)	0.0760 (13)	
H14	0.0692	0.4065	0.4263	0.091*	
C15	0.1775 (4)	0.4089 (4)	0.3168 (2)	0.0598 (10)	
H15	0.1078	0.4720	0.2950	0.072*	
C16	0.5018 (4)	0.6286 (3)	0.24344 (19)	0.0491 (8)	
H16A	0.4059	0.6184	0.2833	0.074*	
H16B	0.4633	0.6481	0.1933	0.074*	
H16C	0.5702	0.6961	0.2516	0.074*	
C17	0.8713 (5)	0.0822 (3)	0.37310 (19)	0.0630 (10)	
H17A	0.8236	0.1665	0.3629	0.076*	
H17B	0.9934	0.0900	0.3682	0.076*	
C18	0.8310 (4)	0.0101 (3)	0.31241 (19)	0.0455 (8)	
C19	0.8622 (4)	−0.0031 (3)	0.17869 (17)	0.0411 (7)	
C20	0.7930 (5)	0.0703 (3)	0.1207 (2)	0.0652 (10)	
H20	0.7735	0.1555	0.1237	0.078*	
C21	0.7519 (6)	0.0231 (4)	0.0590 (2)	0.0734 (12)	
H21	0.7064	0.0745	0.0189	0.088*	
C22	0.7787 (4)	−0.1018 (3)	0.05717 (19)	0.0540 (9)	
C23	0.8398 (4)	−0.1771 (3)	0.11459 (18)	0.0435 (8)	
H23	0.8512	−0.2631	0.1127	0.052*	
C24	0.8858 (4)	−0.1294 (3)	0.17617 (17)	0.0378 (7)	
C25	0.9562 (4)	−0.2199 (3)	0.23510 (17)	0.0388 (7)	
C26	1.0948 (4)	−0.1857 (3)	0.27359 (18)	0.0399 (7)	
C27	1.1021 (4)	−0.2291 (3)	0.3501 (2)	0.0531 (9)	
H31	1.0170	−0.2814	0.3778	0.064*	0.50
C28	1.2290 (6)	−0.1986 (4)	0.3868 (2)	0.0709 (11)	
H28	1.2299	−0.2281	0.4396	0.085*	
C29	1.3531 (5)	−0.1262 (4)	0.3474 (3)	0.0747 (13)	
H29	1.4411	−0.1053	0.3729	0.090*	
C30	1.3528 (4)	−0.0830 (3)	0.2716 (3)	0.0655 (11)	
H30	1.4407	−0.0336	0.2437	0.079*	
C32	1.0045 (5)	0.1643 (3)	0.2218 (2)	0.0575 (10)	
H32A	0.9343	0.2371	0.2294	0.086*	
H32B	1.0555	0.1722	0.1684	0.086*	
H32C	1.0916	0.1578	0.2552	0.086*	
N1	0.6014 (3)	0.5132 (2)	0.24675 (13)	0.0349 (6)	
N2	0.7239 (4)	0.3326 (3)	−0.03396 (17)	0.0622 (9)	
N3	0.9025 (3)	0.0527 (2)	0.24041 (14)	0.0408 (6)	
N4	0.7393 (4)	−0.1532 (4)	−0.00907 (19)	0.0699 (9)	
O1	0.7643 (3)	0.3733 (2)	0.30914 (13)	0.0539 (6)	
O2	0.6212 (9)	0.2889 (10)	−0.0650 (6)	0.0682 (10)	0.503 (11)
O3	0.8746 (8)	0.3465 (9)	−0.0608 (4)	0.0682 (10)	0.503 (11)
O2A	0.5898 (8)	0.3148 (10)	−0.0579 (6)	0.0682 (10)	0.497 (11)
O3A	0.8581 (8)	0.3038 (9)	−0.0666 (5)	0.0682 (10)	0.497 (11)
O4	0.2145 (3)	0.4335 (3)	0.15219 (15)	0.0671 (7)	
O5	0.7432 (3)	−0.0798 (2)	0.32668 (13)	0.0598 (7)	
O6	0.6964 (13)	−0.0882 (7)	−0.0642 (5)	0.0802 (18)	0.503 (11)
O7	0.775 (2)	−0.2652 (7)	−0.0057 (5)	0.0802 (18)	0.503 (11)
O6A	0.6291 (12)	−0.0950 (8)	−0.0452 (5)	0.0802 (18)	0.497 (11)
O7A	0.7985 (19)	−0.2503 (8)	−0.0264 (6)	0.0802 (18)	0.497 (11)
O8	0.9024 (3)	−0.32484 (19)	0.24960 (14)	0.0583 (7)	
F1	0.5148 (2)	0.20503 (17)	0.26090 (12)	0.0662 (6)	
F2	0.9848 (4)	−0.3000 (3)	0.39096 (18)	0.0501 (9)	0.50
F2A	1.2279 (7)	−0.0727 (5)	0.1659 (4)	0.1118 (19)	0.50
Cl1	0.7252 (8)	0.4779 (4)	0.45561 (19)	0.0750 (4)	0.432 (5)
Cl2	0.8013 (6)	0.0204 (5)	0.46559 (17)	0.0750 (4)	0.432 (5)
Cl1A	0.7034 (6)	0.4317 (3)	0.45940 (14)	0.0750 (4)	0.568 (5)
Cl2A	0.7300 (5)	0.0317 (4)	0.45918 (13)	0.0750 (4)	0.568 (5)
C31	1.2222 (4)	−0.1128 (3)	0.2367 (2)	0.0509 (9)	
H31A	1.2206	−0.0810	0.1843	0.061*	0.50

### Atomic displacement parameters (Å^2^)

**Table:**

	*U*^11^	*U*^22^	*U*^33^	*U*^12^	*U*^13^	*U*^23^
C1	0.053 (2)	0.063 (2)	0.0410 (18)	0.0030 (17)	−0.0029 (16)	−0.0080 (16)
C2	0.0333 (17)	0.0402 (18)	0.0450 (18)	−0.0049 (14)	−0.0034 (14)	−0.0045 (14)
C3	0.0358 (16)	0.0265 (15)	0.0444 (17)	0.0022 (12)	−0.0076 (14)	−0.0023 (13)
C4	0.0337 (17)	0.0350 (17)	0.0520 (19)	−0.0020 (13)	−0.0042 (14)	−0.0010 (14)
C5	0.0420 (18)	0.0380 (17)	0.0484 (19)	0.0056 (14)	0.0035 (15)	0.0015 (15)
C6	0.054 (2)	0.0348 (17)	0.0411 (17)	0.0102 (14)	−0.0026 (16)	−0.0008 (14)
C7	0.051 (2)	0.0329 (17)	0.0482 (18)	0.0036 (14)	−0.0151 (16)	−0.0074 (14)
C8	0.0362 (17)	0.0332 (16)	0.0460 (17)	0.0030 (12)	−0.0099 (14)	−0.0080 (13)
C9	0.0365 (18)	0.0419 (18)	0.060 (2)	0.0009 (14)	−0.0116 (16)	−0.0168 (16)
C10	0.0318 (17)	0.0420 (18)	0.058 (2)	−0.0080 (14)	−0.0018 (15)	−0.0150 (15)
C11	0.043 (2)	0.0416 (19)	0.066 (2)	−0.0086 (15)	0.0026 (17)	−0.0086 (17)
C12	0.071 (3)	0.058 (2)	0.068 (3)	−0.008 (2)	0.001 (2)	0.009 (2)
C13	0.083 (3)	0.085 (3)	0.064 (3)	−0.021 (3)	0.014 (3)	−0.004 (2)
C14	0.061 (3)	0.087 (3)	0.076 (3)	−0.003 (2)	0.017 (2)	−0.022 (3)
C15	0.040 (2)	0.068 (2)	0.072 (3)	−0.0051 (17)	0.0019 (18)	−0.017 (2)
C16	0.050 (2)	0.0418 (19)	0.058 (2)	0.0097 (15)	−0.0119 (17)	−0.0141 (16)
C17	0.080 (3)	0.053 (2)	0.060 (2)	0.0080 (19)	−0.011 (2)	−0.0182 (18)
C18	0.0444 (19)	0.0406 (19)	0.052 (2)	0.0103 (15)	−0.0063 (16)	−0.0103 (15)
C19	0.0424 (18)	0.0344 (17)	0.0465 (18)	−0.0006 (13)	−0.0052 (15)	−0.0070 (14)
C20	0.093 (3)	0.0361 (19)	0.071 (2)	0.0108 (18)	−0.032 (2)	−0.0048 (18)
C21	0.107 (3)	0.053 (2)	0.065 (2)	0.007 (2)	−0.042 (2)	0.001 (2)
C22	0.061 (2)	0.054 (2)	0.050 (2)	−0.0052 (17)	−0.0132 (18)	−0.0119 (17)
C23	0.0443 (18)	0.0379 (17)	0.0496 (19)	−0.0034 (14)	−0.0060 (15)	−0.0104 (15)
C24	0.0353 (16)	0.0313 (16)	0.0463 (17)	−0.0012 (12)	−0.0038 (14)	−0.0057 (13)
C25	0.0376 (17)	0.0305 (16)	0.0481 (18)	−0.0015 (13)	−0.0031 (14)	−0.0069 (13)
C26	0.0368 (17)	0.0294 (16)	0.0551 (19)	0.0012 (13)	−0.0084 (15)	−0.0099 (14)
C27	0.058 (2)	0.048 (2)	0.057 (2)	0.0092 (17)	−0.0190 (18)	−0.0144 (17)
C28	0.084 (3)	0.067 (3)	0.072 (3)	0.020 (2)	−0.036 (2)	−0.024 (2)
C29	0.057 (3)	0.070 (3)	0.115 (4)	0.014 (2)	−0.039 (3)	−0.049 (3)
C30	0.040 (2)	0.052 (2)	0.110 (4)	−0.0059 (17)	−0.006 (2)	−0.031 (2)
C32	0.070 (2)	0.0360 (18)	0.067 (2)	−0.0078 (16)	−0.0020 (19)	−0.0130 (16)
N1	0.0324 (13)	0.0309 (13)	0.0445 (14)	0.0010 (10)	−0.0107 (11)	−0.0106 (11)
N2	0.084 (2)	0.0527 (19)	0.0444 (18)	0.0192 (17)	0.0021 (17)	−0.0048 (14)
N3	0.0432 (15)	0.0307 (13)	0.0497 (15)	−0.0001 (11)	−0.0049 (12)	−0.0106 (11)
N4	0.074 (2)	0.081 (3)	0.061 (2)	−0.0025 (19)	−0.0255 (18)	−0.0142 (19)
O1	0.0547 (14)	0.0482 (14)	0.0569 (14)	0.0132 (11)	−0.0104 (12)	−0.0023 (11)
O2	0.0908 (18)	0.065 (4)	0.0550 (16)	0.0240 (17)	−0.0022 (13)	−0.0412 (18)
O3	0.0908 (18)	0.065 (4)	0.0550 (16)	0.0240 (17)	−0.0022 (13)	−0.0412 (18)
O2A	0.0908 (18)	0.065 (4)	0.0550 (16)	0.0240 (17)	−0.0022 (13)	−0.0412 (18)
O3A	0.0908 (18)	0.065 (4)	0.0550 (16)	0.0240 (17)	−0.0022 (13)	−0.0412 (18)
O4	0.0392 (14)	0.091 (2)	0.0759 (17)	0.0017 (13)	−0.0212 (13)	−0.0155 (15)
O5	0.0565 (15)	0.0591 (15)	0.0610 (15)	−0.0140 (12)	0.0073 (12)	−0.0096 (12)
O6	0.088 (4)	0.103 (2)	0.057 (3)	0.002 (3)	−0.022 (3)	−0.026 (2)
O7	0.088 (4)	0.103 (2)	0.057 (3)	0.002 (3)	−0.022 (3)	−0.026 (2)
O6A	0.088 (4)	0.103 (2)	0.057 (3)	0.002 (3)	−0.022 (3)	−0.026 (2)
O7A	0.088 (4)	0.103 (2)	0.057 (3)	0.002 (3)	−0.022 (3)	−0.026 (2)
O8	0.0678 (16)	0.0332 (12)	0.0758 (16)	−0.0164 (11)	−0.0232 (13)	0.0017 (11)
F1	0.0638 (13)	0.0448 (11)	0.0829 (15)	0.0077 (9)	0.0073 (11)	−0.0042 (10)
F2	0.056 (2)	0.055 (2)	0.0343 (18)	−0.0175 (18)	−0.0003 (17)	0.0099 (16)
F2A	0.095 (4)	0.092 (4)	0.139 (5)	−0.020 (3)	0.016 (4)	−0.012 (4)
Cl1	0.0919 (13)	0.0855 (10)	0.0464 (5)	0.0205 (15)	−0.0146 (5)	−0.0069 (6)
Cl2	0.0919 (13)	0.0855 (10)	0.0464 (5)	0.0205 (15)	−0.0146 (5)	−0.0069 (6)
Cl1A	0.0919 (13)	0.0855 (10)	0.0464 (5)	0.0205 (15)	−0.0146 (5)	−0.0069 (6)
Cl2A	0.0919 (13)	0.0855 (10)	0.0464 (5)	0.0205 (15)	−0.0146 (5)	−0.0069 (6)
C31	0.049 (2)	0.0389 (19)	0.061 (2)	−0.0036 (15)	0.0027 (18)	−0.0052 (17)

### Geometric parameters (Å, °)

**Table:**

C1—C2	1.503 (4)	C17—H17B	0.9900
C1—Cl1	1.694 (6)	C18—O5	1.213 (4)
C1—Cl1A	1.825 (5)	C18—N3	1.359 (4)
C1—H1A	0.9900	C19—C20	1.382 (5)
C1—H1B	0.9900	C19—C24	1.398 (4)
C2—O1	1.217 (4)	C19—N3	1.420 (4)
C2—N1	1.354 (4)	C20—C21	1.371 (5)
C3—C4	1.385 (4)	C20—H20	0.9500
C3—C8	1.398 (4)	C21—C22	1.383 (5)
C3—N1	1.424 (4)	C21—H21	0.9500
C4—C5	1.373 (4)	C22—C23	1.353 (5)
C4—H4	0.9500	C22—N4	1.463 (5)
C5—C6	1.374 (5)	C23—C24	1.387 (4)
C5—H5	0.9500	C23—H23	0.9500
C6—C7	1.376 (4)	C24—C25	1.491 (4)
C6—N2	1.465 (4)	C25—O8	1.224 (3)
C7—C8	1.378 (4)	C25—C26	1.484 (4)
C7—H7	0.9500	C26—C31	1.365 (4)
C8—C9	1.502 (4)	C26—C27	1.386 (4)
C9—O4	1.214 (4)	C27—C28	1.372 (5)
C9—C10	1.480 (5)	C27—H31	0.9500
C10—C15	1.385 (4)	C28—C29	1.357 (6)
C10—C11	1.390 (5)	C28—H28	0.9500
C11—C12	1.359 (5)	C29—C30	1.366 (6)
C11—F1	1.361 (4)	C29—H29	0.9500
C12—C13	1.377 (6)	C30—C31	1.375 (5)
C12—H12	0.9500	C30—H30	0.9500
C13—C14	1.370 (6)	C32—N3	1.473 (4)
C13—H13	0.9500	C32—H32A	0.9800
C14—C15	1.383 (5)	C32—H32B	0.9800
C14—H14	0.9500	C32—H32C	0.9800
C15—H15	0.9500	N2—O2	1.212 (7)
C16—N1	1.476 (4)	N2—O3A	1.221 (6)
C16—H16A	0.9800	N2—O2A	1.256 (7)
C16—H16B	0.9800	N2—O3	1.260 (6)
C16—H16C	0.9800	N4—O6	1.208 (7)
C17—C18	1.512 (5)	N4—O7A	1.218 (7)
C17—Cl2	1.717 (5)	N4—O7	1.245 (7)
C17—Cl2A	1.817 (4)	N4—O6A	1.272 (6)
C17—H17A	0.9900	C31—H31A	0.9500
			
C2—C1—Cl1	117.8 (3)	N3—C18—C17	114.8 (3)
C2—C1—Cl1A	108.3 (3)	C20—C19—C24	119.6 (3)
C2—C1—H1A	107.9	C20—C19—N3	118.2 (3)
Cl1—C1—H1A	107.9	C24—C19—N3	122.2 (3)
Cl1A—C1—H1A	99.7	C21—C20—C19	121.5 (3)
C2—C1—H1B	107.9	C21—C20—H20	119.2
Cl1—C1—H1B	107.9	C19—C20—H20	119.2
Cl1A—C1—H1B	124.8	C20—C21—C22	117.9 (4)
H1A—C1—H1B	107.2	C20—C21—H21	121.1
O1—C2—N1	122.4 (3)	C22—C21—H21	121.1
O1—C2—C1	122.5 (3)	C23—C22—C21	122.0 (3)
N1—C2—C1	115.0 (3)	C23—C22—N4	119.4 (3)
C4—C3—C8	119.9 (3)	C21—C22—N4	118.6 (3)
C4—C3—N1	120.3 (3)	C22—C23—C24	120.4 (3)
C8—C3—N1	119.7 (3)	C22—C23—H23	119.8
C5—C4—C3	120.3 (3)	C24—C23—H23	119.8
C5—C4—H4	119.8	C23—C24—C19	118.5 (3)
C3—C4—H4	119.8	C23—C24—C25	116.1 (3)
C4—C5—C6	118.8 (3)	C19—C24—C25	125.4 (3)
C4—C5—H5	120.6	O8—C25—C26	119.9 (3)
C6—C5—H5	120.6	O8—C25—C24	119.2 (3)
C5—C6—C7	122.3 (3)	C26—C25—C24	120.9 (2)
C5—C6—N2	119.8 (3)	C31—C26—C27	116.2 (3)
C7—C6—N2	117.8 (3)	C31—C26—C25	123.0 (3)
C8—C7—C6	118.8 (3)	C27—C26—C25	120.8 (3)
C8—C7—H7	120.6	C28—C27—C26	121.7 (4)
C6—C7—H7	120.6	C28—C27—H31	119.1
C7—C8—C3	119.7 (3)	C26—C27—H31	119.1
C7—C8—C9	117.4 (3)	C29—C28—C27	119.7 (4)
C3—C8—C9	122.9 (3)	C29—C28—H28	120.1
O4—C9—C10	120.9 (3)	C27—C28—H28	120.1
O4—C9—C8	119.3 (3)	C30—C29—C28	120.7 (4)
C10—C9—C8	119.8 (3)	C30—C29—H29	119.7
C15—C10—C11	116.9 (3)	C28—C29—H29	119.7
C15—C10—C9	119.4 (3)	C29—C30—C31	118.3 (4)
C11—C10—C9	123.7 (3)	C29—C30—H30	120.8
C12—C11—F1	117.7 (3)	C31—C30—H30	120.8
C12—C11—C10	123.7 (3)	N3—C32—H32A	109.5
F1—C11—C10	118.5 (3)	N3—C32—H32B	109.5
C11—C12—C13	117.6 (4)	H32A—C32—H32B	109.5
C11—C12—H12	121.2	N3—C32—H32C	109.5
C13—C12—H12	121.2	H32A—C32—H32C	109.5
C14—C13—C12	121.1 (4)	H32B—C32—H32C	109.5
C14—C13—H13	119.4	C2—N1—C3	120.1 (2)
C12—C13—H13	119.4	C2—N1—C16	123.0 (2)
C13—C14—C15	120.0 (4)	C3—N1—C16	116.5 (2)
C13—C14—H14	120.0	O2—N2—O3A	105.4 (5)
C15—C14—H14	120.0	O3A—N2—O2A	122.1 (4)
C10—C15—C14	120.4 (4)	O2—N2—O3	124.8 (4)
C10—C15—H15	119.8	O2A—N2—O3	137.8 (7)
C14—C15—H15	119.8	O2—N2—C6	126.2 (5)
N1—C16—H16A	109.5	O3A—N2—C6	126.5 (5)
N1—C16—H16B	109.5	O2A—N2—C6	111.2 (4)
H16A—C16—H16B	109.5	O3—N2—C6	108.9 (5)
N1—C16—H16C	109.5	C18—N3—C19	119.0 (2)
H16A—C16—H16C	109.5	C18—N3—C32	122.9 (3)
H16B—C16—H16C	109.5	C19—N3—C32	117.6 (2)
C18—C17—Cl2	115.6 (3)	O6—N4—O7A	110.7 (7)
C18—C17—Cl2A	107.1 (3)	O6—N4—O7	125.0 (5)
C18—C17—H17A	108.4	O7A—N4—O6A	120.9 (6)
Cl2—C17—H17A	108.4	O7—N4—O6A	126.4 (9)
Cl2A—C17—H17A	96.2	O6—N4—C22	121.7 (5)
C18—C17—H17B	108.4	O7A—N4—C22	123.0 (6)
Cl2—C17—H17B	108.4	O7—N4—C22	112.7 (6)
Cl2A—C17—H17B	127.7	O6A—N4—C22	116.0 (5)
H17A—C17—H17B	107.4	C26—C31—C30	123.3 (4)
O5—C18—N3	122.3 (3)	C26—C31—H31A	118.4
O5—C18—C17	122.8 (3)	C30—C31—H31A	118.4
			
Cl1—C1—C2—O1	5.3 (5)	C20—C19—C24—C25	−178.9 (3)
Cl1A—C1—C2—O1	−9.9 (4)	N3—C19—C24—C25	−0.9 (4)
Cl1—C1—C2—N1	−176.5 (3)	C23—C24—C25—O8	−35.0 (4)
Cl1A—C1—C2—N1	168.3 (2)	C19—C24—C25—O8	144.5 (3)
C8—C3—C4—C5	1.2 (4)	C23—C24—C25—C26	142.3 (3)
N1—C3—C4—C5	−175.7 (3)	C19—C24—C25—C26	−38.2 (4)
C3—C4—C5—C6	1.9 (4)	O8—C25—C26—C31	139.5 (3)
C4—C5—C6—C7	−2.4 (5)	C24—C25—C26—C31	−37.7 (4)
C4—C5—C6—N2	−179.3 (3)	O8—C25—C26—C27	−39.4 (4)
C5—C6—C7—C8	−0.2 (5)	C24—C25—C26—C27	143.4 (3)
N2—C6—C7—C8	176.7 (3)	C31—C26—C27—C28	1.1 (5)
C6—C7—C8—C3	3.4 (4)	C25—C26—C27—C28	−179.9 (3)
C6—C7—C8—C9	−175.2 (3)	C26—C27—C28—C29	−1.4 (6)
C4—C3—C8—C7	−3.9 (4)	C27—C28—C29—C30	0.2 (6)
N1—C3—C8—C7	173.1 (3)	C28—C29—C30—C31	1.3 (6)
C4—C3—C8—C9	174.7 (3)	O1—C2—N1—C3	−2.6 (4)
N1—C3—C8—C9	−8.4 (4)	C1—C2—N1—C3	179.2 (2)
C7—C8—C9—O4	−42.2 (4)	O1—C2—N1—C16	−175.3 (3)
C3—C8—C9—O4	139.2 (3)	C1—C2—N1—C16	6.5 (4)
C7—C8—C9—C10	136.9 (3)	C4—C3—N1—C2	−61.7 (4)
C3—C8—C9—C10	−41.7 (4)	C8—C3—N1—C2	121.3 (3)
O4—C9—C10—C15	−39.3 (4)	C4—C3—N1—C16	111.4 (3)
C8—C9—C10—C15	141.7 (3)	C8—C3—N1—C16	−65.6 (3)
O4—C9—C10—C11	140.2 (3)	C5—C6—N2—O2	174.7 (8)
C8—C9—C10—C11	−38.9 (4)	C7—C6—N2—O2	−2.3 (9)
C15—C10—C11—C12	−1.4 (5)	C5—C6—N2—O3A	12.5 (8)
C9—C10—C11—C12	179.1 (3)	C7—C6—N2—O3A	−164.5 (7)
C15—C10—C11—F1	176.8 (3)	C5—C6—N2—O2A	−172.9 (6)
C9—C10—C11—F1	−2.7 (4)	C7—C6—N2—O2A	10.0 (7)
F1—C11—C12—C13	−179.0 (3)	C5—C6—N2—O3	−6.4 (6)
C10—C11—C12—C13	−0.8 (5)	C7—C6—N2—O3	176.6 (5)
C11—C12—C13—C14	1.6 (6)	O5—C18—N3—C19	5.5 (5)
C12—C13—C14—C15	−0.3 (7)	C17—C18—N3—C19	−175.3 (3)
C11—C10—C15—C14	2.8 (5)	O5—C18—N3—C32	177.7 (3)
C9—C10—C15—C14	−177.7 (3)	C17—C18—N3—C32	−3.1 (4)
C13—C14—C15—C10	−2.1 (6)	C20—C19—N3—C18	120.9 (3)
Cl2—C17—C18—O5	6.0 (5)	C24—C19—N3—C18	−57.1 (4)
Cl2A—C17—C18—O5	−13.0 (4)	C20—C19—N3—C32	−51.8 (4)
Cl2—C17—C18—N3	−173.1 (3)	C24—C19—N3—C32	130.2 (3)
Cl2A—C17—C18—N3	167.8 (3)	C23—C22—N4—O6	−174.4 (7)
C24—C19—C20—C21	−2.3 (6)	C21—C22—N4—O6	5.8 (8)
N3—C19—C20—C21	179.6 (3)	C23—C22—N4—O7A	−20.9 (10)
C19—C20—C21—C22	1.3 (6)	C21—C22—N4—O7A	159.2 (10)
C20—C21—C22—C23	1.6 (6)	C23—C22—N4—O7	−2.8 (9)
C20—C21—C22—N4	−178.6 (3)	C21—C22—N4—O7	177.3 (8)
C21—C22—C23—C24	−3.3 (5)	C23—C22—N4—O6A	154.2 (7)
N4—C22—C23—C24	176.8 (3)	C21—C22—N4—O6A	−25.7 (8)
C22—C23—C24—C19	2.2 (4)	C27—C26—C31—C30	0.4 (5)
C22—C23—C24—C25	−178.3 (3)	C25—C26—C31—C30	−178.6 (3)
C20—C19—C24—C23	0.6 (4)	C29—C30—C31—C26	−1.6 (5)
N3—C19—C24—C23	178.6 (3)		

### Hydrogen-bond geometry (Å, °)

**Table:**

*D*—H···*A*	*D*—H	H···*A*	*D*···*A*	*D*—H···*A*
C32—H32A···O1	0.98	2.48	3.383 (4)	154.
C29—H29···O5^i^	0.95	2.50	3.194 (5)	130.
C28—H28···Cl1A^ii^	0.95	2.74	3.543 (6)	143.
C17—H17A···O1	0.99	2.38	3.348 (4)	165.
C16—H16B···O2^iii^	0.98	2.47	3.45 (1)	176.
C7—H7···O7^iv^	0.95	2.37	3.31 (2)	173.

Symmetry codes: (i) *x*+1, *y*, *z*; (ii) −*x*+2, −*y*, −*z*+1; (iii) −*x*+1, −*y*+1, −*z*; (iv) −*x*+1, −*y*, −*z*.
